# *Elizabethkingia anophelis* and Association with Tap Water and Handwashing, Singapore

**DOI:** 10.3201/eid2409.171843

**Published:** 2018-09

**Authors:** Chee-Fu Yung, Matthias Maiwald, Liat H. Loo, Han Y. Soong, Chin B. Tan, Phaik K. Lim, Ling Li, Natalie WH Tan, Chia-Yin Chong, Nancy Tee, Koh C. Thoon, Yoke H. Chan

**Affiliations:** KK Women’s and Children’s Hospital, Singapore.

**Keywords:** *Elizabethkingia*, *Elizabethkingia* *anophelis*, pediatrics, intensive care unit, ICU, aerator, water, alcohol handrub, handwashing, outbreak, bacteria, Singapore, tap water

## Abstract

We report an *Elizabethkingia anophelis* case cluster associated with contaminated aerators and tap water in a children’s intensive care unit in Singapore in 2017. We demonstrate a likely transmission route for *E. anophelis* to patients through acquisition of the bacteria on hands of healthcare workers via handwashing.

*Elizabethkingia anophelis* is an emergent pathogen first described from midgut specimens of the *Anopheles gambiae* mosquito (*1*). To date, there have been 2 reported confirmed *E. anophelis* outbreaks in humans. One occurred in an adult critical care unit in Singapore; the second was a large community outbreak in the United States (Wisconsin, Michigan, and Illinois) (*2**–**5*). Water sources have been identified to harbor members of the genus *Elizabethkingia*, but the source of the community outbreak in the United States remains unknown (*3*,*6*). Effective interventions for outbreak control and transmission routes of *E. anophelis* remain unclear (*3*).

KK Women’s and Children’s Hospital (KKH) is the single largest public tertiary-care specialist women’s and children’s hospital in Singapore. The Children’s Intensive Care Unit (CICU) is a 16-bed unit that provides advanced monitoring and therapeutic technologies for critical pediatric cases. On May 30, 2017, an alert was triggered due to the detection of 3 patients with *Elizabethkingia* spp. within 13 days in the unit. The incidence rate of the cluster, 2.87/1,000 bed-days, was ≈4 times higher than the average rate in the previous 5 years, 0.63/1,000 bed-days (2012 through 2016). Initially, the strains were reported as *E. meningoseptica,* but subsequent testing confirmed the cluster to be associated with *E. anophelis*. We conducted an epidemiologic investigation to identify the source of the cluster. We also conducted a pragmatic experiment to test our hypothesis that *E. anophelis* could be transmitted by healthcare workers during handwashing with water contaminated with *E. anophelis.*

## The Study

We collated clinical and epidemiologic data using a standardized spreadsheet for all patients testing positive for *Elizabethkingia* species in the KKH CICU in 2017. We also performed environmental sampling on all tap outlets and sinks in the clinical areas. For each tap, we swabbed the aerator and collected a water sample for culture. The water source of KKH has no supplemental treatments and meets WHO guidelines for drinking-water quality (*7*). To test our transmission hypothesis, we had 2 volunteer nurses place their hands on agar plates at 3 stages: before handwashing; after handwashing with chlorhexidine soap (4% wt/vol chlorhexidine gluconate; Microshield 4 Chlorhexidine Surgical Handwash, Schülke; Norderstedt, Germany) and tap water from the tap outlet in CICU known to be positive for *E. anopheles*; and finally after hand hygiene using alcohol-based hand rub (ABHR) (70% vol/vol ethanol and 0.5% wt/vol chlorhexidine gluconate; Microshield Handrub; Schülke).

We tested samples using matrix-assisted laser desorption/ionization time-of-flight (MALDI-TOF) mass spectrometry (VITEK MS; bioMérieux, Marcy-l’Étoile, France). We retested all samples positive for *Elizabethkingia* spp. by using 16S rDNA PCR: we extracted bacterial DNA and amplified 16S rDNA using primers 27f and 1492r (*8*). We performed sequencing using standard protocols and used BLAST (https://blast.ncbi.nlm.nih.gov/Blast.cgi) for comparison with database sequences. 

The 3 cluster cases were the only patients positive for *Elizabethkingia* species in the CICU in 2017. All were detected from blind bronchial sampling (BBS) via endotracheal tube (ETT). (Table 1) Patient 3’s isolate was confirmed as *E. anophelis.* Unfortunately, the samples from the first 2 cases were not available for follow-up confirmatory testing. The patients were 2.8 months, 4.9 months, and 4.8 years of age, and all had significant underlying medical conditions. The average number of days in CICU before detection of *Elizabethkingia* species was 36 (range 11–66). None of the patients had been moved since admission, and 2 were cared for in single rooms.

**Table 1:** Characteristics of *Elizabethkingia* cases in children admitted to the Children’s Intensive Care Unit, KK Women’s and Children’s Hospital, Singapore, May 2017*

Category	Patient 1	Patient 2	Patient 3
Sample date	2017 May 15	2017 May 22	2017 May 28
Sample type	ETT, BBS	ETT, BBS	ETT, BBS
Bacterial identification			
MALDI-TOF mass spectrometry	*E. meningoseptica*	*E. meningoseptica*	*E. meningoseptica*
16S rDNA	Isolate not available	Isolate not available	*E. anophelis*
Sex	M	F	F
Age, mo	4.9	2.8	57.9
Preterm birth	No	No	No
Underlying clinical condition	Duodenal atresia; small atrial septal heart defect	Pulmonary atresia; Large ventral septal heart defect; large patent ductus arteriosus	Thoracic tumor
Outcome	Discharged	Discharged	Deceased
Days in hospital	11	83	33
CICU bed type	Single room	4-bed cubicle	Single room
Other beds used	No	No	No
Antimicrobial drug treatment within 72 h before detection	Piperacillin/tazobactam, ceftriaxone	Clindamycin	Piperacillin/tazobactam
History of immunosuppressive medication	No	No	Yes (chemotherapy)
On ECMO at time of detection	Yes	No	Yes

*ETT, endotracheal tube; CICU, Children’s Intensive Care Unit; ECMO, extracorporeal membrane oxygenation; ETT, endotracheal tube; MALDI-TOF, matrix-assisted laser desorption/ionization time-of-flight.

Of the 27 environmental samples collected from 9 tap outlets or sinks in the unit, 10 samples were positive for *E. anophelis* and 1 positive for *E. meningoseptica.* Only 1 room (single bed) in the unit was negative for *Elizabethkingia* bacteria. All 3 *Elizabethkingia* case-patients’ rooms or cubicles were confirmed positive for *E. anophelis* from their respective tap outlets (aerator or water or both). The tap outlet from 1 cubicle not associated with any of the cases was positive for both *Elizabethkingia* species, *E. meningoseptica* in water and *E. anophelis* in the aerator. The Figure illustrates the spatial distribution of *Elizabethkingia* bacteria detected in tap outlets stratified by aerator, water, or sinks in the unit.

**Figure:**
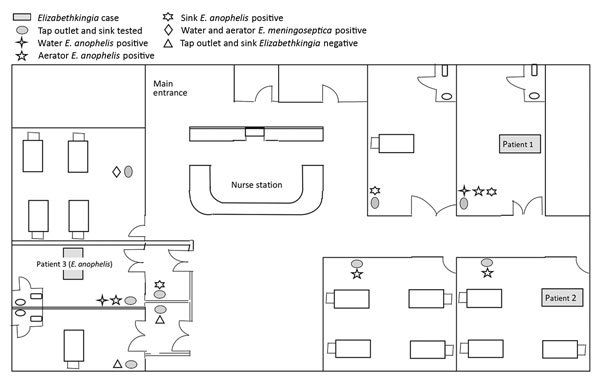
Spatial distribution of *Elizabethkingia* isolates by location (patients, tap water, aerators, and sinks) in children's intensive care unit, KK Women’s and Children’s Hospital, Singapore, May 2017.

Our transmission experiment found that 1 staff member (staff B) acquired *E. anophelis* on her hands after handwashing (Table 2). After hand hygiene using ABHR, both staff members had no detectable microbial growth on their hands.

**Table 2:** Potential transmission route of *E. anophelis* via handwashing for 2 hospital staff, Children’s Intensive Care Unit, KK Women’s and Children’s Hospital, Singapore, May 2017

Procedure	Hands culture result	
	Staff A	Staff B
Before handwashing	Coagulase-negative *Staphylococcus* sp.	Coagulase-negative *Staphylococcus* sp.
After handwashing with chlorhexidine soap	Coagulase-negative *Staphylococcus* sp.	*E. anophelis*
After use of alcohol-based hand rub	No growth	No growth

Upon detection of the case cluster, we reinforced standard precautions, specifically hand hygiene compliance, and implemented environmental and patient-care equipment cleaning. We had all aerators permanently removed from the tap outlets in the CICU following confirmation of *Elizabethkingia* bacteria. The water from all 5 tap outlets previously found to be positive for *Elizabethkingia* bacteria in aerator or water was negative upon repeat testing after the intervention. We also recommended prioritizing hand hygiene using ABHR over handwashing unless hands were visibly soiled. All staff were reminded not to dispose of body fluids from patients into sinks used for handwashing because this was previously identified to be associated with *Elizabethkingia* tap colonization (*2*). In addition, we ended the use of tap water for patient care and allowed only sterile water. After these interventions, no additional cases of *Elizabethkingia* occurred in the unit for >4 months.

## Conclusions

We report a confirmed *E. anophelis* case cluster affecting infants and children in the CICU of a pediatric hospital. Our investigation identified the likely source of *E. anophelis* to be tap outlets with aerators. We confirmed that removal of the aerators was effective in eliminating *E. anophelis* from tap water sources. We also demonstrated a likely transmission route for *E. anophelis* to patients through acquisition of the bacteria on hands of healthcare workers via handwashing. Subsequent use of ABHR was effective in eliminating the acquired *E. anophelis* from workers’ hands.

Although 2 patients’ isolates were not available for confirmatory testing, we detected *E. anophelis* in the tap outlets where they were cared for, suggesting that the *Elizabethkingia* species detected in their samples was highly likely to be *E. anophelis.* Isolates were initially misidentified as *E. meningoseptica* by MALDI-TOF mass spectrometry because *E. anophelis* was not represented in our routine database and only present in research databases of MALDI-TOF mass spectrometry systems (*9*). This discrepancy means that *E. anophelis* is probably overlooked in most diagnostic microbiology laboratories. There is a clinical need to differentiate these species in light of observations that *E. anophelis* infections tend to be more severe and associated with more deaths than are *E. meningoseptica* infections (*10*).

We showed how handwashing, despite the use of chlorhexidine soap, is a possible vehicle of transmission for *E. anophelis* from an affected tap outlet via the hands of healthcare workers to patients. Perinatal transmission of *E. anophelis* was previously documented to have occurred from a mother with chorioamnionitis to her neonate (*11*). We confirmed that hand hygiene using ABHR was effective in removing *E. anophelis* from hands of healthcare workers, which has implications for infection control. Although current hand hygiene guidelines prioritize ABHR over handwashing when hands are not visibly soiled, there is no requirement to perform ABHR in addition to handwashing (*12*). Therefore, most staff consider handwashing as complying with hand hygiene requirements. Our findings support using ABHR as the primary hand-hygiene method in clinical care, especially in critical care units and in outbreak situations involving waterborne organisms such as *E. anophelis*.
